# Combinatory strategy for characterizing and understanding the ethanol synthesis pathway in cyanobacteria cell factories

**DOI:** 10.1186/s13068-015-0367-z

**Published:** 2015-11-21

**Authors:** Guodong Luan, Yunjing Qi, Min Wang, Zhimin Li, Yangkai Duan, Xiaoming Tan, Xuefeng Lu

**Affiliations:** Key Laboratory of Biofuels, Qingdao Institute of Bioenergy and Bioprocess Technology, Chinese Academy of Sciences, No. 189 Songling Road, Qingdao, 266101 China; Shandong Provincial Key Laboratory of Synthetic Biology, Qingdao Institute of Bioenergy and Bioprocess Technology, Chinese Academy of Sciences, No. 189 Songling Road, Qingdao, 266101 China; Shandong Provincial Key Laboratory of Energy Genetics, Qingdao Institute of Bioenergy and Bioprocess Technology, Chinese Academy of Sciences, No. 189 Songling Road, Qingdao, 266101 China; Qingdao University of Science and Technology, Qingdao, 266061 China; University of Chinese Academy of Sciences, Beijing, 100049 China; College of Bioscience and Bioengineering, Jiangxi Agricultural University, Nanchang, 330045 Jiangxi China

**Keywords:** *In vitro* reconstitution, Steady-state analysis, Ethanol, Cyanobacteria, Metabolic engineering

## Abstract

**Background:**

Photosynthetic production of chemicals and fuels by recycling CO_2_ in cyanobacteria is a promising solution facing energy shortage and resource declination. Ethanol is an attractive and demonstrative biofuel product, and ethanol synthesis in cyanobacteria has been achieved by assembling of a pathway consisting of pyruvate decarboxylase (*PDCzm*) and alcohol dehydrogenase II (*slr1192*). For enabling more powerful ethanol photosynthetic production, an optimized and balanced catalyzing route was required. In this work, we provided a paradigm for systematically characterizing and optimizing the PDCzm-slr1192 pathway from engineered cyanobacteria strains, combining *in vitro* reconstitution, genetic engineering and feeding-cultivation.

**Results:**

We reconstituted the PDCzm-slr1192 pathway *in vitro* and performed specific titration assays for enzymes, substrates, cofactors, and metal ions. In the *in vitro* system, *K*_50_ of PDCzm was 0.326 μM, with a *V*_max_ of 2.074 μM/s; while for slr1192, the values were 0.109 μM and 1.722 μM/s, respectively. Titration response discrepancy indicated that PDCzm rather than slr1192 was the rate-limiting factor for ethanol synthesis. In addition, a 4:6 concentration ratio of PDCzm-slr1192 would endow the reaction with a maximal specific catalytic activity. Titration assays for other components were also performed. *K*_*m*_ values for NADPH, pyruvate, TPP, Mg^2+^ and acetaldehyde were 0.136, 6.496, 0.011, 0.104, and 0.393 mM, respectively. We further constructed *Synechocystis* mutant strains with diverse PDCzm-slr1192 concentrations and ratios, and compared the growth and ethanol synthesis performances. The results revealed that activities of PDCzm indeed held control over the ethanol generation capacities. We performed pyruvate-feeding treatment with the newly developed Syn-YQ4 strain, and confirmed that improvement of pyruvate supply would direct more carbon flow to ethanol formation.

**Conclusions:**

We systematically characterized and optimized the PDCzm-slr1192 pathway in engineered cyanobacteria for ethanol production. Information gained from *in vitro* monitoring and genetic engineering revealed that for further enhancing ethanol synthesis capacities, PDCzm activities needed enhancement, and the PDCzm-slr1192 ratio should be improved and held to about 1:1.5. Considering actual metabolites concentrations of cyanobacteria cells, enhancing pyruvate supply was also a promising strategy for further updating the current ethanol photosynthetic cell factories.

**Electronic supplementary material:**

The online version of this article (doi:10.1186/s13068-015-0367-z) contains supplementary material, which is available to authorized users.

## Background

Globally increasing energy demands and irreversibly declining fossil resources are driving the development of efficient and sustainable production routes for chemicals and fuels [1]. Photosynthetic cell factories directly channeling solar energy and CO_2_ into aimed products are generally accepted as one of solutions [2–4]. Cyanobacteria have displayed great potentials to be a promising photosynthetic chassis, with the characteristics of simple structure, rapid growth, and convenient genetic manipulations [5, 6]. Assembling, inserting, and fine-tuning of the heterologous and endogenous enzymes in cyanobacteria has enabled photosynthetic generation of multiple products, including alcohols, ketones, alka(e)nes, organic acids, and sugars [7–13].

Ethanol is the first reported and most representative cyanobacteria-based photosynthetic biofuel product. In 1999, Deng and Coleman endowed *Synechococcus* sp. PCC7942 with ethanol synthesis capacities by introduction of a 2-step metabolic pathway from *Zymomonas mobilis*, consisting of pyruvate decarboxylase (*PDCzm* encoded) and alcohol dehydrogenase II (*ADHzm* encoded) [8], which has also been widely applied in many other ethanol-producing microbial cell factories [14–16]. *ADHzm* was further replaced with a native alcohol dehydrogenase *slr1192* from *Synechocystis* sp. PCC6803 showing improved NADPH-preference [17] and catalytic efficiency [17, 18] in cyanobacteria. Ever since, many strategies and methods have been adopted for efficiently rerouting the carbon fixed in Calvin cycle to ethanol formation in engineered cyanobacteria strains, including deletion or weakening of competitive pathways, improvement of photosynthesis activities, strengthening of precursor supplies, and engineering of ethanol-tolerance, while catalytic activities of the core pathway (PDC-ADH) proved to be the most essential factor determining ethanol synthesis capacities [18–23]. For developing more powerful ethanol photosynthetic cell factories, an optimized and balanced PDC-ADH catalytic process would be the premise, which requires systematic characterization and comprehensive understanding of the metabolic pathway.

In this work, we adopted a systematic strategy combining *in vitro* reconstitution, genetic engineering and feeding-cultivation to characterize and understand the *PDCzm*-*slr1192* pathway in ethanol synthesis strains of *Synechocystis* sp. PCC6803 (*Synechocystis* hereafter). For monitoring and unveiling the kinetic characteristics and system properties of the conversion process from pyruvate to ethanol, we reconstituted the *PDCzm*-*slr1192* pathway *in vitro* and performed quantitative titrations for each specific component, including enzymes, substrate, and cofactors. Accurate contribution assessments for each factor of the ethanol synthesis process indicated that improving the PDCzm-slr1192 concentration ratio and enhancing pyruvate supply might be promising engineering strategies for promoting ethanol photosynthetic production. For validation, we regulated the *PDCzm* and *slr1192* copy numbers in *Synechocystis* to disturb the enzyme concentration ratios, and took pyruvate-feeding strategies to enhance the substrate supply. Calculations of enzymatic activity, cell growth, ethanol synthesis, and carbon partition ratio of the *Synechocystis* strains matched well with the predictions from *in vitro* system, indicating that our results could serve as useful guidance for updating the current ethanol photosynthetic cell factories.

## Results

### *In vitro* reconstitution and quantitative titrations of PDCzm and slr1192 for ethanol synthesis

**Fig. 1:**
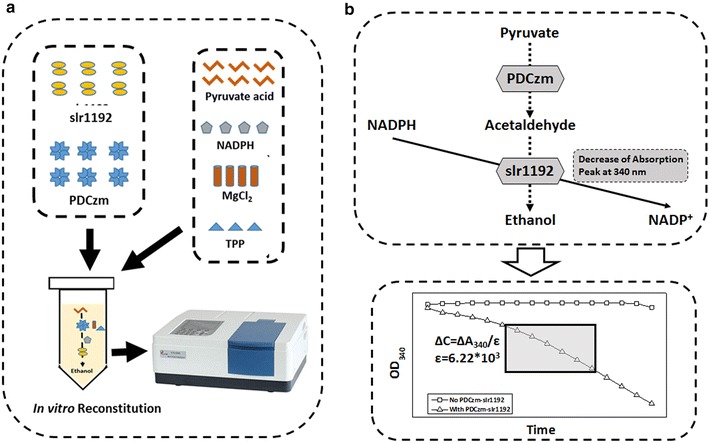
*In vitro* reconstitution of the PDCzm-slr1192 pathway. **a** Overview of the *in vitro* reconstitution process. **b** Catalytic process of PDCzm-slr1192 and the mechanisms for velocity calculation. Equal moles of NADPH would be consumed by slr1192 during the ethanol synthesis process, and NADPH has an absorbance peak at 340 nm, so the consumption speed of NADPH could be used to calculate the reaction velocities

As shown in Fig. 1, the conversion process in engineered *Synechocystis* strains from pyruvate to ethanol was catalyzed by two enzymes, PDCzm catalyzing the decarboxylation of pyruvate to acetaldehyde, which would be further converted to ethanol by slr1192. During the process, equal moles of NADPH would be consumed by slr1192 when ethanol was generated, so the consumption speeds of NADPH could be measured to evaluate the reaction velocities.

*PDCzm* and *slr1192* were expressed under T7 promoter in *Escherichia coli* BL21 (DE3) and purified individually. As an initial reference system, 0.05 μM PDC and 0.05 μM slr1192 were added to a 500 μl reaction system containing 100 mM Tris–HCl, 10 mM pyruvates, 0.2 mM NADPH, 10 mM MgCl_2_ and 0.1 mM TPP. Absorbance of the system at 340 nm was measured with a Beckman-coulter DU-800 spectrophotometer, and the decreasing speed of NADPH was converted to the reaction velocities based on the absorptivity of NADPH, 6.22 × 10^3^ l/mol/cm. For the initial reference system, the velocity was 0.452 ± 0.04 μM/s.

**Fig. 2:**
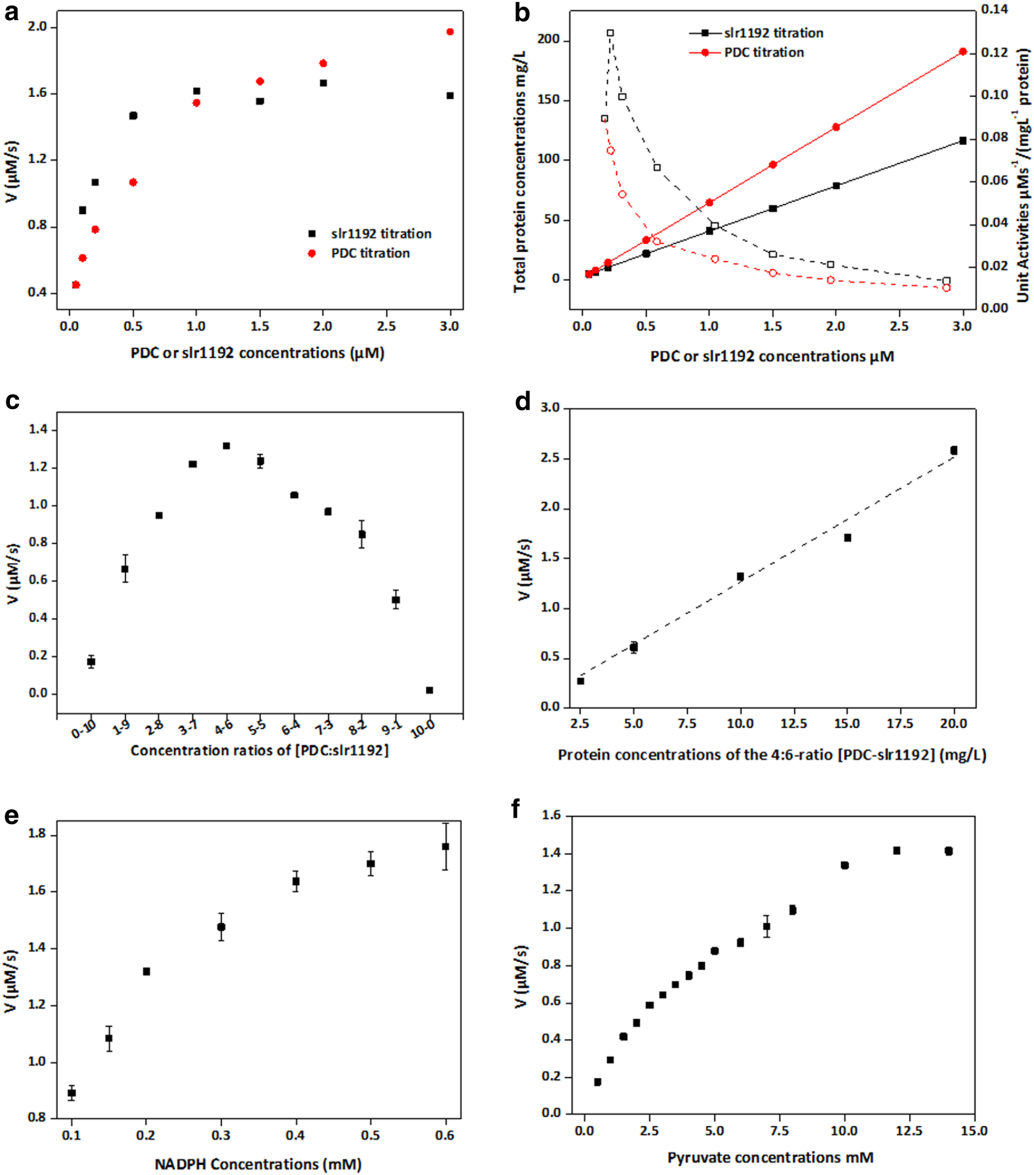
Quantitative titration and optimization of the PDCzm-slr1192 pathway based on the *in vitro* reconstitution system. **a** In slr1192 titration system (*black*), PDCzm concentrations were fixed at 0.05 μΜ, while in PDCzm titration system (*red*), slr1192 concentrations were fixed at 0.05 μΜ. **b** Total protein concentrations (*closed*) and unit activities (*open*) dynamics of the PDCzm titration (*circle*) and slr1192 titration (*square*) systems. **c** Regulation of PDCzm and slr1192 ratios in the *in vitro* reconstitution systems with fixed total protein concentrations (10 mg/l). **d** Titration of the optimized (PDCzm-slr1192) unit with a concentration ratio of 4:6. The single unit indicated a concentration ratio of 4:6 between PDCzm and slr1192, while serial concentrations of such a unit were added to the *in vitro* system. **e** Titration of NADPH as cofactor in the optimized *in vitro* system. **f** Titration of pyruvate as substrate in the optimized *in vitro* system

We further performed *in vitro* titrations for PDCzm and slr1192 to assess the specific contributions from each enzyme and identify the rate-limiting step for ethanol synthesis. Concentrations of one enzyme were increased to 0.1, 0.2, 0.5, 1, 1.5, 2, and 3 μM, while that of the other one was kept at 0.05 μM. As shown in Fig. 2a, titrations of PDCzm and slr1192 revealed markedly different responses. According to a previously reported calculation method [24], initial reaction velocities versus enzyme concentrations plots were fitted with the Michaelis–Menten model, while the substrate were replaced with enzyme (*V* = *V*_max_ × *E*/(*K*_50_ + *E*), here *K*_*m*_ value for substrate was replaced with *K*_50_ for enzyme). *K*_50_ value of PDCzm was about 0.326 μM, with a *V*_max_ value of 2.074 μM/s, while *K*_50_ and *V*_max_ for slr1192 were 0.109 μM and 1.722 μM/s, respectively. The relatively higher *K*_50_ and *V*_max_ of PDCzm comparing with slr1192 indicated that for the initial reference system, PDCzm rather than slr1192 played as the main rate-limiting factor.

We further analyzed the specific activities dynamics of the PDCzm-slr1192 total proteins during the titration processes. As shown in Fig. 2b, for PDCzm titration, with increasing of the total protein concentrations, specific activities of the PDCzm-slr1192 system kept decreasing from 0.089 to 0.01 μM/(s mg/l protein). As for the slr1192 titration, a peak with the highest specific activities [0.13 μM/(s mg/l protein)] appeared when the slr1192 concentration reached 0.1 μM, indicating that the most economical and balanced concentration ratio of PDCzm-slr1192 might be around 1:2.

### Optimization of the PDCzm-slr1192 ratio for the *in vitro* assay system

As for construction of microbial cell factory, improving and optimizing expression levels of the enzymes in the metabolic pathway was always an important strategy; however, concentrations of specific enzymes could not be endlessly increased. A balanced and economical concentration ratios between diverse metabolic components might be a more promising choice for *in vivo* pathway engineering. Thus we regulate the concentration ratios of PDCzm and slr1192 from 0:10 to 10:0 in the *in vitro* system with total protein concentrations of 10 mg/l.

As shown in Fig. 2c, when the concentration ratio of PDCzm-slr1192 reached 4:6 (mole ratio 1:2.5), reaction velocities of the *in vitro* system taking NADPH as the cofactor were maximized. That result perfectly matched the response of slr1192 titration, in which reaction system with a 1:2 mole ratio of PDCzm-slr1192 showed the highest specific activities. We also measured the velocity dynamics for diverse PDCzm-slr1192 concentration ratios in *in vitro* system taking NADH as the cofactor. As shown in Additional file 1: Figure S1, when 0.2 mM NADH rather than NADPH was added to the *in vitro* system, reaction velocities decreased dramatically, due to the low preference of slr1192 for NADH [25]. Meanwhile, the most efficient concentration ratio moved to be 3:7 instead of 4:6, and that might be caused by that the decrease of slr1192 activities in the NADH environments disturbed the previously generated balance. Considering that cellular concentrations of NADPH is about tenfold higher than that of NADH in cyanobacteria [26], the 4:6 ratio was selected to optimize the *in vitro* system.

To evaluate the stability of the optimized concentration ratio, we added a series of concentrations of PDCzm-slr1192 (4:6 ratio) into the *in vitro* system, and calculated the reaction velocity dynamics. Figure 2d showed a nearly linear increase of reaction velocities when total protein concentrations increased from 2.5 to 20 mg/l. These results indicated that as for cyanobacteria engineering for efficient synthesis of ethanol, concentrations of PDCzm and slr1192 should be regulated to be much higher and held to the ratio of about 4:6.

### Substrate, cofactors, and metal ions titrations in the optimized *in vitro* system

The successful catalysis of PDCzm and slr1192 was assisted by several other components, including cofactor supply, substrate supply, and metal ions. So we further quantified contributions and influences of specific factor for the catalytic properties of the PDCzm-slr1192 pathway. As reported above, cofactor types (NADPH and NADH) greatly determined activities of slr1192. In this part, we performed *in vitro* titration to assess the concentration influence from NADPH. Serial concentrations of NADPH, from 0.1 to 0.6 mM, were added to the *in vitro* system with 10 mg/l PDCzm-slr1192 (4:6 ratio as previously optimized), and the velocity dynamics versus NADPH concentrations were fitted with a Michaelis–Menten model as applied for enzyme titrations (Fig. 2e). The *K*_*m*_ of NADPH for titration in the *in vitro* system was 0.136 mM, with a *V*_max_ value of 2.211 μM/s.

Pyruvate supply seemed to be of great importance for catalytic properties of the PDCzm-slr1192 pathway. The velocities would be gradually improved to the maximal level when pyruvate concentrations were above 10 mM (Fig. 2f), a level much higher than the actual pyruvate concentrations in cyanobacteria cell (about 20–100 μM) [7, 27]. *K*_*m*_ and *V*_max_ values calculated from the pyruvate titration assay were 6.496 mM and 2.064 μM/s, respectively (Table 1).

**Table 1:** *In vitro* titration fit values of PDCzm, slr1192, NADPH, pyruvate, TPP, Mg^2+^ ion and acetaldehyde

Components	*K*_50_ */K*_*m*_	*V*_max_ (μM/s)
PDCzm	0.326 ± 0.071 μM	2.074 ± 0.120
slr1192	0.109 ± 0.014 μM	1.722 ± 0.045
NADPH	0.136 ± 0.013 mM	2.211 ± 0.085
Pyruvate	6.496 ± 0.505 mM	2.064 ± 0.104
TPP	0.011 ± 0.002 mM	1.405 ± 0.054
Mg^2+^	0.104 ± 0.017 mM	1.329 ± 0.043
Acetaldehyde	0.393 ± 0.130 mM	3.538 ± 0.228

PDCzm and slr1192 titrations were performed in an *in vitro* system containing 10 mM pyruvates, 0.2 mM NADPH, 10 mM MgCl_2_, 0.1 mM TPP, 0.05 μM PDCzm or slr1192. NADPH, pyruvate, TPP, Mg^2+^ and acetaldehyde titrations were performed in an optimized system containing 10 mg/l PDCzm-slr1192 with a concentration ratio of 4:6

We also regulated concentrations of acetaldehyde, the process metabolite, TPP and Mg^2+^, another two important factors regulating properties of PDCzm, and calculated the activities dynamics (Additional file 1: Figures S2 and S3). As shown in Table 1, *K*_*m*_ values of acetaldehyde, TPP and Mg^2+^ were 0.393, 0.011 and 0.104 mM, respectively.

### Genetic engineering of *Synechocystis* for regulation of PDCzm-slr1192 ratios

**Fig. 3:**
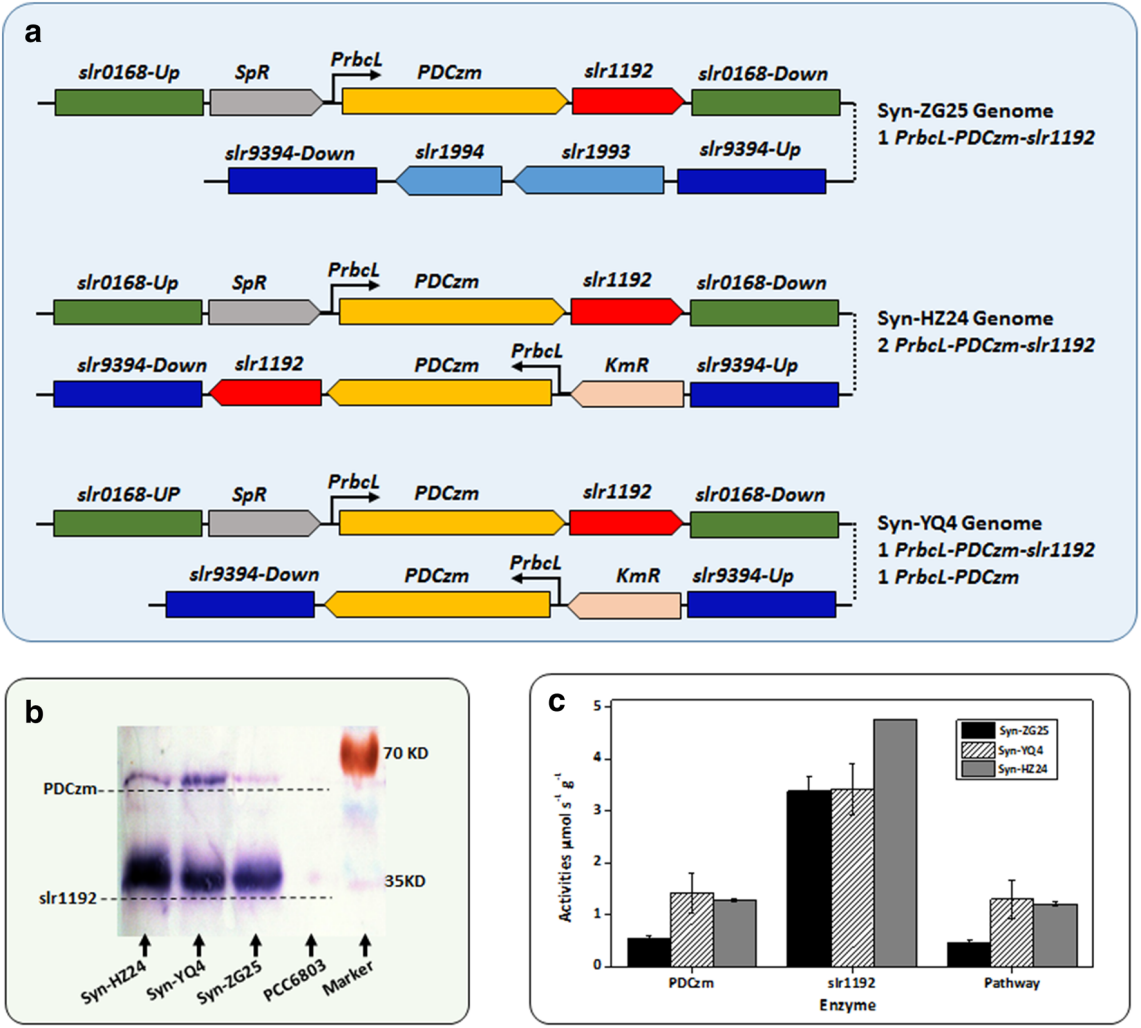
Genetic engineering of *Synechocystis* for regulation of PDCzm-slr1192 ratios. **a** Genetic modifications on genomes of Syn-ZG25, Syn-HZ24, and Syn-YQ4. *Slr0168*-*UP* and *slr0168*-*Down* up-stream and down-stream homologous regions for PrbcL-PDCzm-slr1192 cassette integration with slr0168 site. *Slr9394*-*Up* and *slr9394*-*Down* up-stream and down-stream homologous regions for PrbcL-PDCzm-slr1192 cassette integration with slr1993-slr1994 site. *SpR* spectinomycin resistance gene. *KmR* kanamycin resistance gene. **b** Western blot analysis for PDCzm and slr1192 concentration in PCC6803, Syn-ZG25, Syn-HZ24, and Syn-YQ4. **c** Pyruvate decarboxylase, alcohol dehydrogenase, and PDCzm-slr1192 pathways activities calculations for crude enzyme of Syn-ZG25, Syn-HZ24, and Syn-YQ4

*In vitro* reconstitution and quantitative titrations of PDCzm and slr1192 indicated that conversion process from pyruvate to acetaldehyde was the rate-limiting step of the ethanol synthesis pathway, thus increasing the PDCzm-slr1192 concentration ratio should be a target for pathway optimization. For confirmation, we constructed *Synechocystis* strains with different *PDCzm*-*slr1192* copy number ratios to generate diverse concentration ratios. Previously we have developed two *Synechocystis* mutant strains for ethanol synthesis, Syn-ZG25 (ZG25 hereafter) containing 1 *PrbcL*-*PDC*-*slr1192* cassette copy and Syn-HZ24 (HZ24 hereafter) containing 2 *PrbcL*-*PDC*-*slr1192* cassette copies. In vivo concentrations of PDCzm and slr1192 were both significantly improved in HZ24 comparing with ZG25, while the concentration ratio was relatively constant [18]. To disturb the PDCzm-slr1192 concentration ratio, we constructed a strain containing 1 PrbcL-PDC-slr1192 copy (on the slr0168 site, a neutral site on chromosome of *Synechocystis* sp. PCC6803) and 1 PrbcL-PDC copy (on the slr1993-slr1994 site, encoding acetyl CoA acetyltransferase and 3-ketoacyl-ACP reductase, essential for PHB synthesis in *Synechocystis* sp. PCC6803, slr9394 hereafter). The new strain was developed from ZG25, and termed as Syn-YQ4 (YQ4 hereafter), as shown in Fig. 3a.

Western blot analysis (Fig. 3b) revealed that comparing with ZG25 and HZ24, PDCzm-slr1192 concentration ratio in YQ4 was significantly improved. Slr1192 concentrations in YQ4 and ZG25 were on the same level, much lower than that of HZ24, while PDCzm concentration in YQ4 was even higher than that in HZ24 (as shown in Additional file 1: Figure S4).

Catalytic activities calculations further confirmed the results of protein concentration analysis. As shown in Fig. 3c, alcohol dehydrogenase activities of HZ24 crude enzyme was 4.76 μmol/s/g, about 40 % higher than that of ZG25 and YQ4 (3.39 and 3.41 μmol/s/g, respectively). While activities of pyruvate decarboxylase in HZ24 and YQ4 crude enzymes were quite close to each other (1.28 and 1.41 μmol/s/g, respectively), more than twofold than that of ZG25 (0.55 μmol/s/g). PDCzm activity patterns matched well with the PDCzm-slr1192 pathway activity analyses, velocities of HZ24 and YQ4 crude enzymes on the same level (1.29 and 1.30 μmol/s/g, respectively), while much higher than that of ZG25 (0.46 μmol/s/g).

**Fig. 4:**
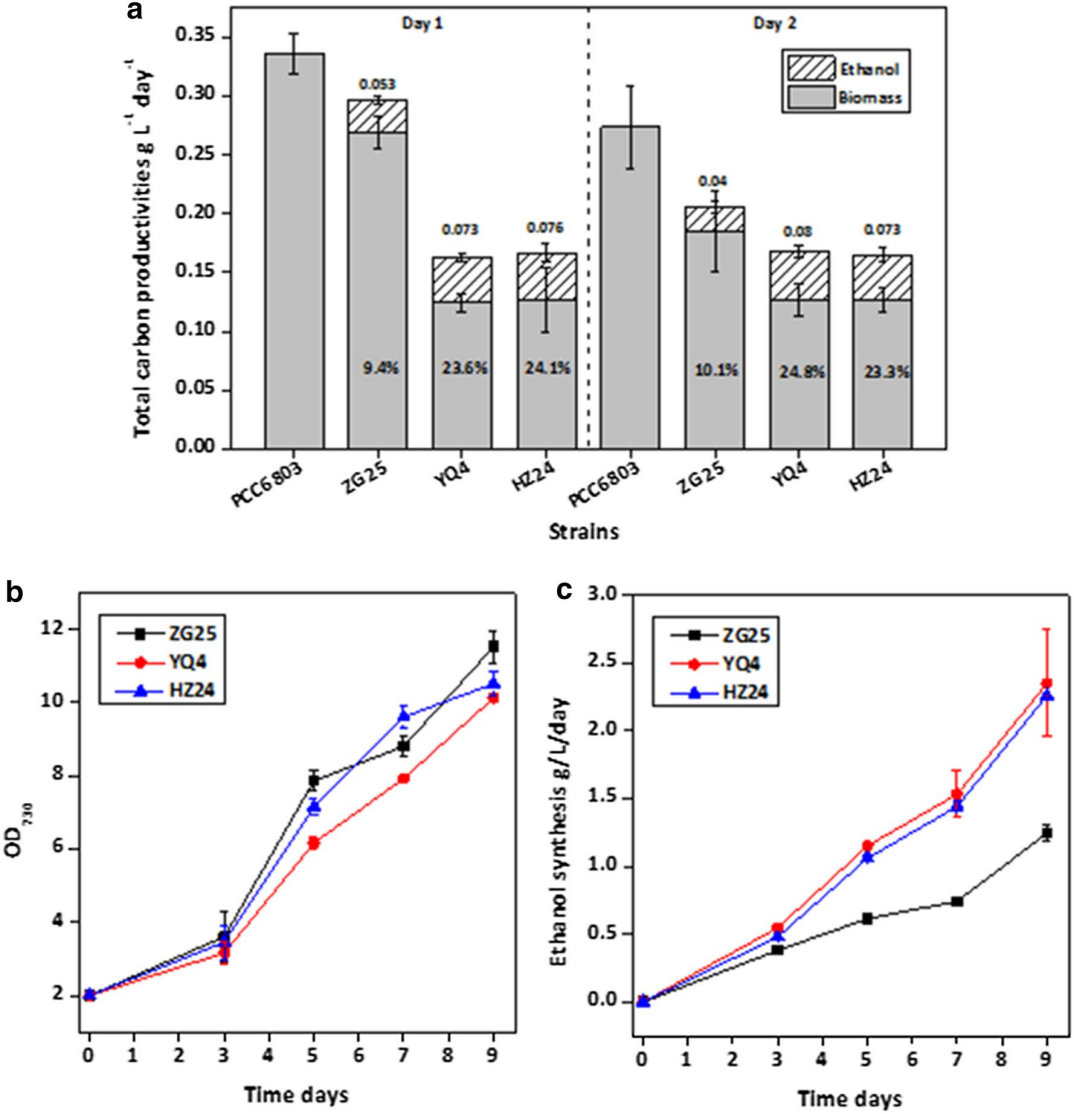
Growth and ethanol production assay of strains with diverse PDCzm-slr1192 ratios. **a** Total carbon productivities and carbon partitioning ratio to ethanol of PCC6803, Syn-ZG25, Syn-HZ24, and Syn-YQ4, taking NaHCO_3_ as carbon source. Carbon distributed to ethanol and biomass was calculated by measurement of ethanol concentration and OD730. *Numbers in the bars* percent of total fixed carbon distributed to ethanol, *numbers above the bars* the actual ethanol concentration synthesized by the strain (g/l/day). **b** Growth and **c** ethanol production of the three strains cultivated in column photo-reactor system

For accurate evaluation of ethanol synthesis capacities of the *Synechocystis* mutants holding diverse PDCzm-slr1192 ratios and concentrations, we cultivated the strains in BG11 medium using 50 mM NaHCO_3_ as sole carbon source. As shown in Fig. 4, in 2-day cultivation utilizing NaHCO_3_, over 20 % of the fixed carbon was converted into ethanol in HZ24 and YQ4, while in ZG25 the ratio was about 10 %. Ethanol concentrations in cultures of ZG25, YQ4, and HZ24 were 0.093, 0.153, and 0.15 g/l, respectively. Ethanol synthesis and carbon partitioning ratios indicated that YQ4 strain obtained the same level of ethanol-producing capacities comparing with HZ24, while with much lower slr1192 concentrations and activities.

Growth and ethanol synthesis capacities of the *Synechocystis* mutants holding diverse PDCzm-slr1192 ratios and concentrations were also evaluated with a column photo-reactor system as previously introduced [18]. As shown in Fig. 4b, c, after 9 days of cultivation, ethanol concentrations of the YQ4 and HZ24 cultivation system reached 2.2–2.3 g/l, while that of the ZG25 system was 1.2 g/l. Ethanol production of the YQ4 strain was improved by twofold than that of ZG25.

### Pyruvate-feeding assay of YQ4 strain

As indicated by the *in vitro* titration results, improving pyruvate supply would benefit the ethanol generation capacities of engineered *Synechocystis* strains. Previously it has been discovered that environmental pyruvate could be taken up and metabolized by cyanobacterium *Synechococcus elongatus* PCC7942 [28], thus we applied a convenient strategy to evaluate the influence of increased pyruvate supply by adding pyruvate to the BG11 medium and calculate the biomass and ethanol accumulation.

**Fig. 5:**
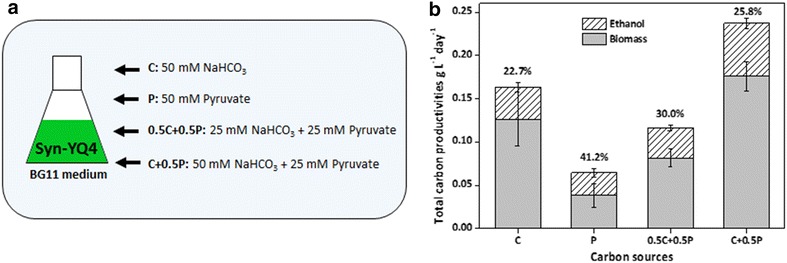
Total carbon productivities and carbon partitioning ratio to ethanol of Syn-YQ4 in BG11 medium supplemented with pyruvate. **a** Concentrations of NaHCO_3_ and pyruvate for different feeding treatments. **b** Carbon accumulated in biomass and ethanol for YQ4 treated with different feeding pattern. *Numbers above the bars* the percent of total carbon distributed to ethanol

As shown in Fig. 5, when 50 mM pyruvate was added into BG11 medium to replace NaHCO_3_ as sole carbon source, carbon partitioning ratio of ethanol in YQ4 strain was sharply improved to 41.2 %, while both biomass and ethanol accumulations were significantly inhibited. When 25 mM pyruvate and 50 mM NaHCO_3_ were simultaneously supplemented into BG11, speeds of biomass accumulation and ethanol synthesis were both improved, with an increased carbon partitioning ratio to ethanol. Results of the YQ4 strain pyruvate-feeding cultivation indicated that redistributing more carbon fixed by cyanobacteria to pyruvate might be useful strategy for enhancing ethanol productivities.

## Discussion

As one of the most attractive and promising technical routes for biofuels production, exploring metabolic engineering principles for cyanobacteria synthesis of ethanol is of great demonstrative significance for development of efficient photosynthetic cell factories. Previously it has been proved that as for cyanobacteria-based production of chemicals and biofuels, metabolic capacities of the core pathways usually imposed the essential control over the product formation rates and carbon partitioning ratios [2, 11, 18, 29, 30]. Focusing on ethanol synthesis in cyanobacteria, the most effective improvement was achieved by increasing copy numbers of *PDCzm*-*slr1192* on chromosomes or high-copy plasmids [18, 19], further confirming that the metabolic pathway activity was the main restricting factor for photosynthetic production of ethanol. However, expression strengths of the catalytic components could not be endlessly increased due to the limitation of genetic manipulation tools and the requirements of the host cells for maintaining basic homeostasis. Thus economical, balanced and efficient metabolic pathways were premises for development of more powerful photosynthetic cellular factories, which requires comprehensive understanding of the catalytic processes. In this work, we provided a paradigm for systematically characterizing and optimizing an essential ethanol synthesis metabolic pathway of engineered cyanobacteria strains, combining *in vitro* reconstitution, genetic engineering and feeding-cultivation.

*In vitro* reconstitution and steady-state analysis is a newly arising strategy for monitoring and unveiling the kinetic characteristics and system properties of metabolic pathways. The information thus gained has facilitated understanding and optimizing *E. coli* fatty acid synthase system [31], farnesene production pathway [32], and cyanobacteria fatty acid synthase system [24]. Comparing with previous *in vitro* reconstitution research, we attempted to optimize enzyme ratios with a constant total concentrations, rather than simply single-factor titration. In our previously developed ethanol synthesis strains ZG25 and HZ24, the *in vivo* concentration ratios of PDCzm to slr1192 were about 1:30–1:40 [18], far away from the most economical ratio of 1:2.5 we identified in this work, indicating that PDCzm activities hold the control over ethanol generation performance. For validation, we constructed a new *Synechocystis* strain YQ4 based on ZG25, with improved PDCzm concentrations. Biomass accumulation and ethanol generation assays revealed that the ethanol synthesis capacities of YQ4 were significantly improved than that of ZG25, while totally comparable with that of HZ24, although the slr1192 concentration was significantly decreased. Information gained from *in vitro* reconstitution and genetic engineering indicated that further enhancing PDCzm concentrations or activities should be the key point for enabling more efficient photosynthetic production of ethanol, which would be benefited from the development of novel expression toolkit in cyanobacteria [33, 34]. Protein engineering should be an alternative strategy. Considering that pyruvate decarboxylase from *Z. mobilis* had the highest reported catalytic activities [35], equipping the cell factory with optimized *PDCzm* obtained from directed evolution or rational designing might be of great significance for accelerating the pyruvate decarboxylation process during ethanol synthesis.

With removed restrictions from metabolic enzymes, reaction velocities of the *in vitro* systems were mainly controlled by substrate supply. As for 10 mg/l PDCzm-slr1192, the reaction velocities would be maximized when initial pyruvate concentrations were above 10 mM, a level far more than normal concentration in cyanobacteria (about 20–100 μM) [7, 27]. In order to explore the influence of increased *in vivo* supply of pyruvate, we added pyruvate into culture medium of the newly obtained *Synecgocystis* strain YQ4. As expected, when pyruvate was added, carbon partitioning ratio of ethanol was sharply increased. However, it is also noteworthy that when pyruvate concentration reached a high level, both cell growth and ethanol synthesis were significantly inhibited, indicating that a balance should be maintained between the carbon source distributed to biomass and chemicals. Pyruvate is an essential cellular central metabolite in cyanobacteria, from which carbon flow was distributed to diverse derivates. Introduction of artificially designed metabolic modulars or pathways oriented with pyruvate have enabled production of diverse chemicals, including lactic acids [7], ethanol [8], 2,3-butanediol [36, 37], acetone [13], isopropanol [10], and butanol [9]. Concentration of the main precursor phosphoenolpyruvate (PEP) was about tenfold higher than that of pyruvate, thus enhancement of conversion from PEP to pyruvate might greatly benefit ethanol production as reported in lactic acid production engineering [27, 29]. Previously it has been proved that knockout of the glycogen synthesis pathway could effectively promote accumulation and excretion of pyruvate [38], thus systematic genetic modifications of cyanobacteria to rewire carbon flow for enhanced pyruvate supply should also be a promising strategy for development of more efficient photosynthetic microbial platforms.

Sufficient and suitable cofactor supply was always a guarantee for the unencumbered chemical production in cyanobacteria [29, 39]. Comparing with the initial photosynthetic cell factory for ethanol production, replacing the NADH-dependent *AdhII* from *Z. mobilis* with the native NADPH-dependent gene *slr1192* significantly improved catalytic activities of the whole pathway [18]. In our *in vitro* titration assays with serial PDCzm-slr1192 concentration ratios from 1:9–7:3, reaction velocities were decreased by over 80 % when taking NADH as cofactor rather than NADPH, indicating the importance of optimizing cofactor preferences in heterogeneous metabolic pathway of cyanobacteria. As for cyanobacteria, photosynthesis played an important role for guaranteeing the rapid turnover from NADP^+^ to NADPH, thus possibly maintaining the NADPH concentration at a favorable level for ethanol production. Engineering or updating the key components or enzymes in photosystem for more efficient supply of NADPH and carbon source could also contribute a lot for biosynthesis in cyanobacteria [40].

## Conclusions

In summary, we combined *in vitro* reconstitution, genetic engineering, and feeding-cultivation for systematically characterizing and optimizing the PDCzm-slr1192 pathways in engineered cyanobacteria strains. The information gained from *in vitro* monitoring and genetic engineering indicated that enhancing PDCzm activities and improving PDCzm-slr1192 concentration ratio to about 1:1.5 should be essential point for further improvement of ethanol generation rates. Taking the actual metabolite concentrations of cyanobacteria cells into consideration, expanding pyruvate supply might also benefit ethanol formation capacities in cyanobacteria.

## Methods

### Chemicals and reagents

Unless noted otherwise, all reagents were purchased from Sigma-Aldrich (USA). *Taq* DNA polymerase and all restriction enzymes were purchased from Fermentas (Canada) or Takara (Japan). The kits used for molecular cloning were from Omega (USA) or Takara (Japan). Oligonucleotides were synthesized and DNA sequencing was performed by Sunnybio (Shanghai, China).

### Strains and plasmids constructions

*Escherichia coli* DH5α was used in this work for plasmid construction and *E. coli* BL21 (DE3) was used as the host for protein expression. Strains Syn-ZG25 and Syn-HZ24 were constructed in our previous work [18], and Syn-YQ4 was constructed by introduction of the plasmid pYQ4 into the *slr9394* site of the strain Syn-ZG25.

For construction of pYQ4 plasmid, up and down homologous recombination arms of *slr9394* were cloned and fused with CK2 (kanamycin resistance gene) and *PrbcL*-*PDC* fragment, respectively, by overlap-PCR. The two fused fragments were finally inserted into pMD18T (TAKARA).

Transformation of plasmid pYQ4 into Syn-ZG25 was performed as the published procedures [41]. Syn-ZG25 was cultivated to exponential phase, and the cells were centrifuged, washed twice with fresh BG11 medium, and finally resuspended to a density of about 1 × 10^9^ cells/ml. Plasmid DNA was added to the cell suspension to a final concentration of about 10 μg/ml. The cell–DNA mixture was incubated for 6 h at 30 °C under luminous intensity of about 50 μE/m^2^/s) and then spread on nitrocellulose filters on BG11 agar plates for 24 h. Finally, the filters were moved to fresh BG11 agar plates containing 25 μg/ml kanamycin and 25 μg/ml spectinomycin. After cultivation for about 10 days, single colonies were separated and cultivated in liquid BG11 for re-checking.

### Protein expression and purification

Plasmid pXT4 for *PDCzm* expression and plasmid pXT113A for *slr1192* expression were constructed in previous work [18]. *E. coli* BL21 (DE3) was transformed with pXT4 and pXT113A, respectively. One single colony of the transformed *E. coli* BL21 (DE3) strain was inoculated in 11 ml LB medium added with 50 μg/ml kanamycin and cultivated for 12 h before being inoculated into 1 l super broth (tryptone 1.2 %, yeast extract 1.4 %, KH_2_PO_4_ 0.38 %, K_2_HPO_4_ 1.25 %, glycerol 0.5 %). The broth was cultivated at 37 °C, 220 rpm for about 2.5 h, till the OD_600_ reached 0.4 and isopropyl-d-thiogalactopyranoside (IPTG) was added to a final concentration of 0.5 mM. The culture is incubated at 16 °C overnight with shaking at 180 rpm.

Cells were harvested by centrifugation, resuspended in binding buffer (20 mM Tris–HCl, 0.5 M NaCl, pH 7.9 for slr1192 and pH 7.5 for PDCzm) and lysed by sonication. The cell lysate was pelleted by centrifugation at 12,000*g*, 4 °C for 30 min. The supernatant was immediately blended with Ni-NTA resin (Novagen), pre-equilibrated with the binding buffer, and agitated at 4 °C for 1 h. The equilibrated system was then transferred into a 5-ml column that was washed sequentially with 25 ml binding buffer, 25 ml washing buffer containing serial concentrations (20, 40, and 80 mM) of imidazole to remove nonspecifically bound proteins, and then 25 ml of the washing buffer (containing 100 mM imidazole for PDCzm and 250 mM for slr1192) to elute the target proteins. The eluted protein was examined by SDS-PAGE and the elution fractions with desired purity were pooled, desalinated with 100 mM Tris–HCl, 0.5 M NaCl (pH 7.5 for PDCzm and pH 7.9 for slr1192), and finally concentrated with Amicon Ultra centrifugal filters (Milipore). Concentrations of the purified proteins were quantified using the Bradford method [42].

### *In vitro* reconstitution and UV kinetic assay of the PDCzm-slr1192 pathway

Initial rates of the ethanol synthesis pathway of PDCzm-slr1192 was quantified using a spectrophotometric NAD(P)H consumption assay, as previously described for PDCzm activity *in vitro* assay [18], while the components and respective concentrations of the *in vitro* system were modified and regulated as required. Slr1192 oxidized one NADPH or NADH per turn over, so the 340 nm absorbance of the reaction system decreased during NAD(P)H to NAD(P)^+^ could be directly related to ethanol synthesis.

For steady-state analysis, an initial *in vitro* system containing 100 mM Tris–HCl, 10 mM MgCl_2_, 0.1 mM thiamine pyrophosphate (TPP), 0.05 μM PDCzm, 0.05 μM slr1192, 0.2 mM NADPH, and 10 mM pyruvate was constructed (pH 7.5), and the total volume was 500 μl. Titrations of PDCzm and slr1192 were performed in the initial system, while for titration of other components, 4 mg/l PDCzm and 6 mg/l slr1192 were added to the reaction as introduced below. Reactions were always initiated by addition of the PDCzm-slr1192 components, and the 340 nm absorbance is measured with a Beckman-coulter DU-800 spectrophotometer. All experiments were repeated for at least 3 times to verify the trends and parametric reproducibility. For PDCzm-slr1192 pathway activities analysis, crude enzymes of the wild-type and engineered *Synechocystis* strains were added into the reaction system instead of purified enzymes.

### Cultivation of *Synechocystis* strains

For growth and ethanol production assay, *Synechocystis* strains were cultivated in BG11 medium added with 50 mM NaHCO_3_, 10 mM TES–NaOH (pH 8.0), and appropriate antibiotics, and incubated with shaking at 30 °C in a incubator with 130 rpm under moderate intensity white-light illumination (about 100 μE/m^2^/s). Cell density and ethanol concentration of in the culture were calculated every 24 h by removing 0.5 ml from the 25 ml total volume. For pyruvate-feeding assay, 25 mM pyruvate plus with 25 mM NaHCO_3_ or 50 mM pyruvate is supplemented into the BG11 medium to replace the 50 mM NaHCO_3_.

Optical densities of the cyanobacteria culture were used for calculation of carbon in biomass. Dry cell weight (gDW) was calculated from OD730 using the value of 0.22 g DW/l/OD730 [28], while carbon content of cells was taken as 51.34 % [28, 43]. Carbon in ethanol was calculated from the concentration of ethanol from the culture. Partitioning ratio of carbon to ethanol was obtained by dividing the carbon in ethanol by the sum of total carbon distributed in ethanol and biomass during the same period.

*Synechocystis* mutant strains were also cultivated in column photo-bioreactors as previously introduced [18]. *Synechocystis* cells grown to the exponential phase were harvested by centrifugation and resuspended to an OD_730_ of 2.0 with 200 ml fresh BG11 medium in column bioreactor (580 mm × 30 mm) with a rubber plug. The cultures in the column photo-bioreactor system were sparged with 5 % CO_2_-air under 37 °C with constant 100 μE/m^2^/s. The rate of gas addition is about 200 ml/min. To measure the ethanol production more accurately, a condenser and a recovery bottle were connected with the column bioreactor due to the volatility of ethanol [18].

### Extraction of *Synechocystis* cell crude enzyme

20 ml *Synechocystis* culture at late exponential growth with an OD730 of about 1.5 was harvested by centrifugation at 4 °C. The resulted cell pellets were resuspended in 2 ml pre-chilled Tris–HCl buffer (50 mM Tris–HCl, pH 8.0) and disrupted with 100 μm glass beads (Sigma). After removing the cell debris and glass beads by 4 °C centrifugation, the supernatants were collected for western blot analysis and crude enzyme activity assay. Protein concentrations of the cell-free extracts were also measured with Bradford method [42].

### Crude enzyme activity assay

Alcohol dehydrogenase and pyruvate decarboxylase activities of the *Synechocystis* mutants cell crude enzymes were analyzed as previously introduced with minor modifications [18]. For alcohol dehydrogenase activity analysis, crude enzyme was added to the reaction buffer containing 100 mM Tris–HCl, 0.2 mM NADPH, and 10 mM acetaldehyde. For pyruvate decarboxylase activity analysis, crude enzyme were added to the reaction buffer containing 100 mM Tris–HCl, 10 mM MgCl_2_, 0.1 mM thiamine pyrophosphate (TPP), 0.2 mM NADPH, 10 mM pyruvate and 400 μM purified slr1192. Reactions were always initiated by addition of crude enzymes, and the 340 nm absorbance is measured with a Beckman-coulter DU-800 spectrophotometer. All experiments were repeated for at least 3 times to verify the trends and parametric reproducibility.

### Ethanol production analysis

Samples of the cyanobacteria culture were centrifuged at 10,000*g* for 10 min, and the supernatants were used for ethanol concentration measurement with an SBA-40c biosensor analyzer (Shandong Academy of Sciences, China) equipped with ethanol oxidase immobilized membrane, as previously introduced [18].

### SDS-PAGE and western blot analysis

The protein samples were analyzed on 12 % SDS-PAGE with a standard procedure and blotted to PVDF membrane, sealed in 5 % nonfat milk-TBST buffer (TBS added with 0.05 % Tween-20) at 4 °C overnight. First, the membrane was incubated with anti-6×His-Tag monoclonal antibodies (from mouse) for 3 h and washed 3 times with TBST (15 min each time). Second, the membrane were incubated with an alkaline phosphatase-linked secondary antibody (donkey anti-mouse) for 1 h and washed 3 times with TBST (15 min each time). Finally, the membrane was colored with BCIP/NBT (Sigma). For expression level comparisons, the results of western blot were calculated with ImageJ.

## Additional file

10.1186/s13068-015-0367-z **Figure S1.** Regulation of PDCzm and slr1192 ratios in the *in vitro* reconstitution systems with fixed total protein concentrations (10 mg/L) taking NADH as cofactor. **Figure S2.** Titrations of TPP and Mg^2+^ for PDCzm-slr1192 pathway in the optimized in vitro reconstitution system containing total protein concentrations (10 mg/L) taking NADPH as cofactor. **Figure S3.** Titration of acetaldehyde for PDCzm-slr1192 pathway in the optimized *in vitro* reconstitution system containing the total protein concentration (10 mg/L) taking NADPH as cofactor. **Figure S4.** Relative expression level of PDCzm and slr1192 in Syn-ZG25, Syn-YQ4 and Syn-HZ24. The concentration of specific protein in ZG25 was set as 100.

## Abbreviations

PDC: pyruvate decarboxylase
PDCzm: pyruvate decarboxylase from *Zymomonas mobilis*
NADPH: reduced nicotinamide adenine dinucleotide phosphate
NADP: nicotinamide adenine dinucleotide phosphate
NADH: reduced nicotinamide adenine dinucleotide
NAD: nicotinamide adenine dinucleotide
TPP: thiamine pyrophosphate
SpR: spectinomycin resistance gene
KmR: kanamycin resistance gene
slr9394: slr1993 and slr1994
