# Supplementary material for: Combinatory strategy for characterizing and understanding the ethanol synthesis pathway in cyanobacteria cell factories
Source: Biotechnol Biofuels. 2015 Nov 21;8:184. doi: 10.1186/s13068-015-0367-z (PMC4654843; doi:10.1186/s13068-015-0367-z)
Supplement: Supplementary file 1 — 10.1186/s13068-015-0367-z Figure S1. Regulation of PDCzm and slr1192 ratios in the in vitro reconstitution systems with fixed total protein concentrations (10 mg/L) taking NADH as cofactor. Figure S2. Titrations of TPP and Mg2+ for PDCzm-slr1192 pathway in the optimized in vitro reconstitution system containing total protein concentrations (10 mg/L) taking NADPH as cofactor. Figure S3. Titration of acetaldehyde for PDCzm-slr1192 pathway in the optimized in vitro reconstitution system containing the total protein concentration (10 mg/L) taking NADPH as cofactor. Figure S4. Relative expression level of PDCzm and slr1192 in Syn-ZG25, Syn-YQ4 and Syn-HZ24. The concentration of specific protein in ZG25 was set as 100. [file 13068_2015_367_MOESM1_ESM.docx]

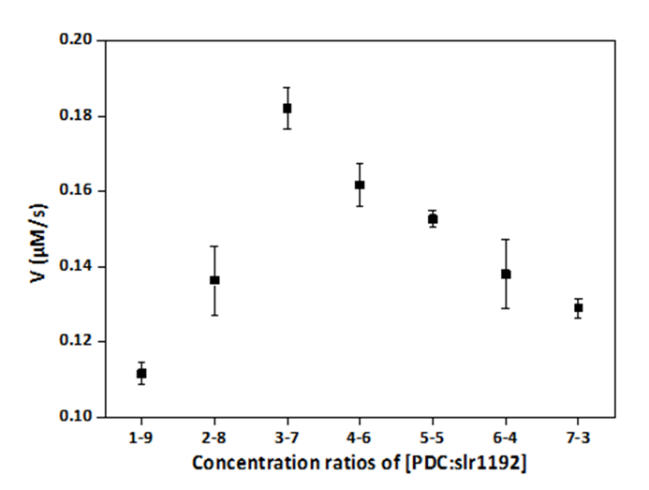


Figure S1. Regulation of PDCzm and slr1192 ratios in the *in vitro* reconstitution systems with the fixed total protein concentration (10 mg/L) taking NADH as cofactor.


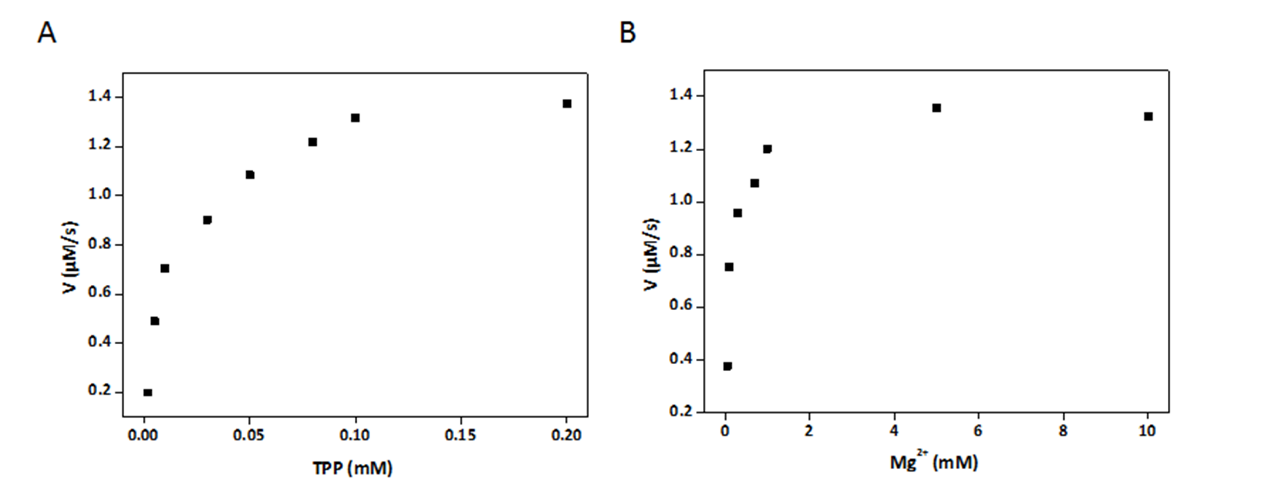


Figure S2. Titrations of TPP (A) and Mg^2+^ (B) for PDCzm-slr1192 pathway in the optimized *in vitro* reconstitution system containing the total protein concentration (10 mg/L) taking NADPH as cofactor.


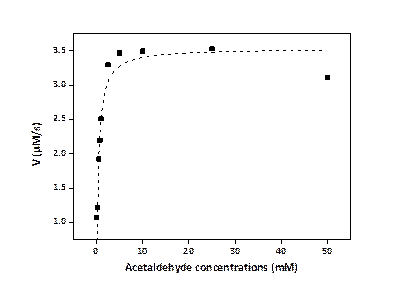


Figure S3. Titration of acetaldehyde for PDCzm-slr1192 pathway in the optimized *in vitro* reconstitution system containing the total protein concentration (10 mg/L) taking NADPH as cofactor.





Figure S4. Relative expression level of PDCzm and slr1192 in Syn-ZG25, Syn-YQ4 and Syn-HZ24. The concentration of specific protein in ZG25 was set as 100.





Figure S5. Optimization of PDCzm-slr1192 ratios in sealed volume with 200 μM pyruvate.
